# Transperitoneal laparoscopic right radical nephrectomy for renal cell carcinoma and end-stage renal disease: a case report

**DOI:** 10.1186/1757-1626-2-200

**Published:** 2009-11-18

**Authors:** Christoforos Kosmıdis, Christoforos Efthimiadis, Georgios Anthimidis, Marios Grigoriou, Kalliopi Vasiliadou, Georgia Ioannidou, Sofia Baka, Epaminondas Fahantidis

**Affiliations:** 1 Department of Surgery, Interbalkan European Medical Center, Thessaloniki, Greece; 2 Department of Radiology, "Panagia" General Hospital, Thessaloniki, Greece; 3 Department of Oncology, Interbalkan European Medical Center, Thessaloniki, Greece; 4 1st Propeudeutic Surgical Clinic of AUTh., AHEPA Hospital, Thessaloniki, Greece

## Abstract

Nephron-sparing surgery (partial nephrectomy) results are similar to those of radical nephrectomy for small (<4 cm) renal tumors. However, in patients with end-stage renal disease, radical nephrectomy emerges as a more efficient treatment for localized renal cell cancer. Laparoscopic radical nephrectomy (LRN) increasingly is being performed. The objective of the present study was to present a case of a patient under hemodialysis who was submitted to LRN for a small renal mass and discuss the current issues concerning this approach. It appears that radical nephrectomy should be the standard treatment in dialysis patients even for small tumors. The laparoscopic technique is associated with acceptable cancer-specific survival and recurrence rate along with shorter hospital stay, less postoperative pain and earlier return to normal activities.

## Introduction

Malignant tumors of kidney account for 3-4% of all new cancer cases each year [1]. Renal cell carcinoma (RCC) comprises 80-85% percent of all primary renal neoplasms [2]. Nephron-sparing surgery (partial nephrectomy) has become the standard treatment for small (<4 cm) renal tumor [3]. However, in patients with end-stage renal disease, radical nephrectomy emerges as an oncologically more sound procedure for localized RCC [4]. Laparoscopic radical nephrectomy (LRN) has been shown to provide equivalent oncologic results for RCC [5-7].

We present herein a case of a patient under hemodialysis who was submitted to LRN for a small renal mass and discuss the current issues concerning this approach.

## Case presentation

**Figure 1:**
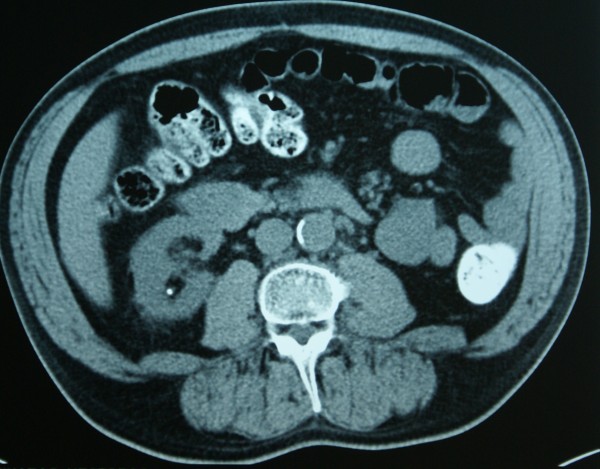
**Abdominal computed tomography (CT) scan showing a calcified 2,5 × 2,7 cm mass at the lower pole of the right kidney**.

**Figure 2:**
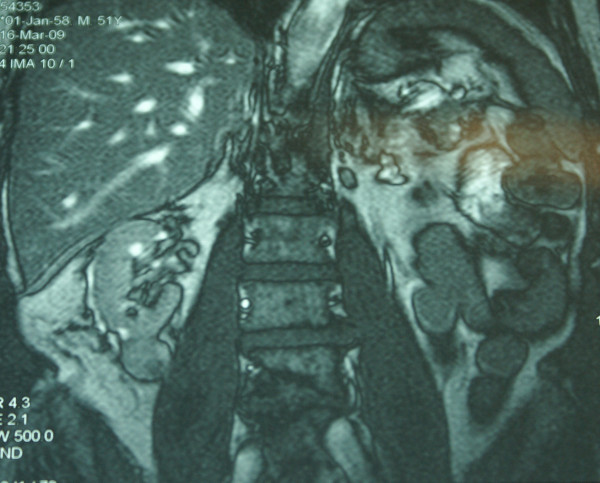
**Magnetic resonance imaging T2 (MRI) scan also showing a 2,5 × 2,7 cm mass at the lower pole of the right kidney and an adenoma of the right adrenal gland**.

**Figure 3:**
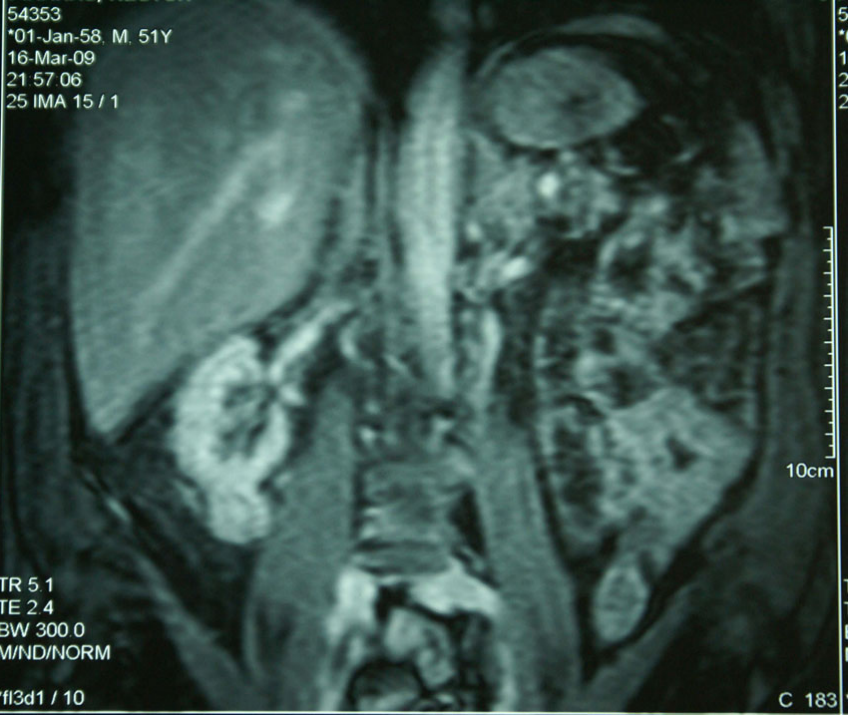
**Magnetic resonance imaging post contrast (MRI) scan also showing a 2,5 × 2,7 cm mass at the lower pole of the right kidney and an adenoma of the right adrenal gland**.

**Figure 4:**
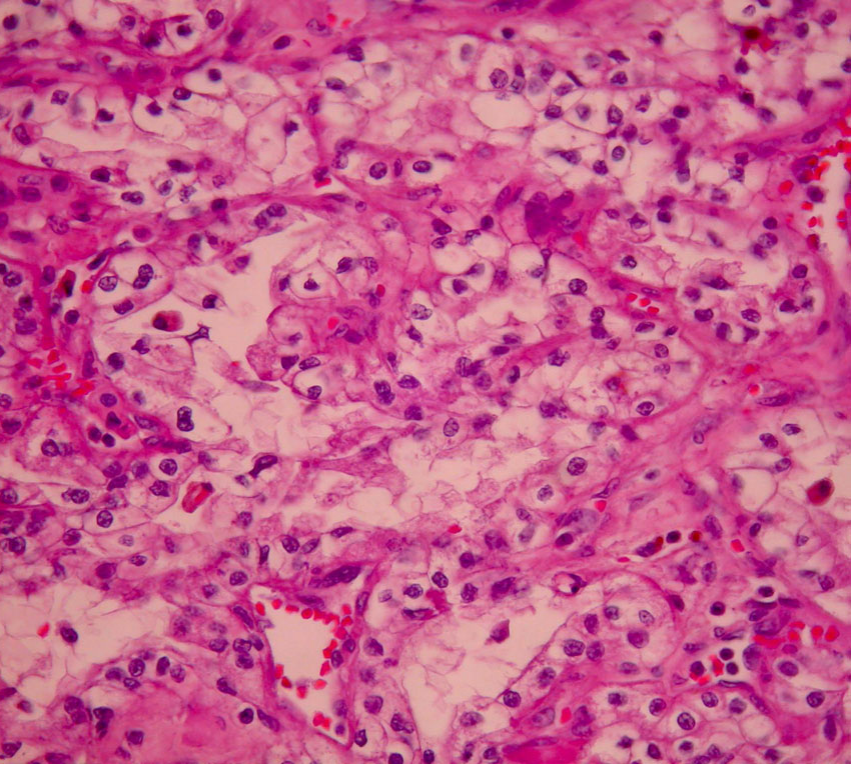
**Classical features of clear renal cell carcinoma (H+E×400)**.

**Figure 5:**
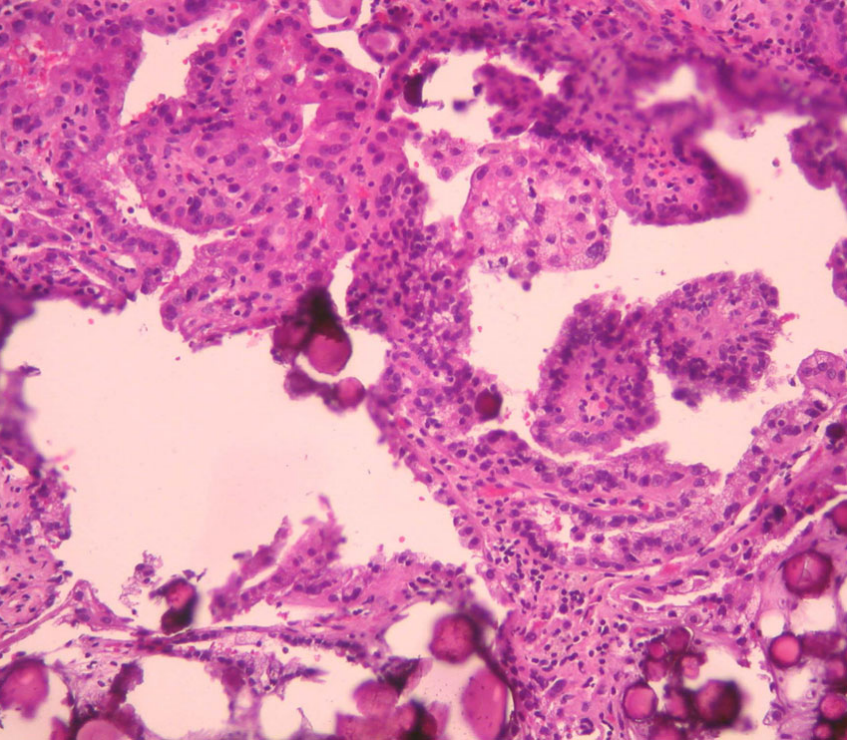
**Classical features of small papillary adenoma of the adrenal gland (H+E×200)**.

A 51-year-old Greek man presented with fatigue. Laboratory tests, including complete blood cell count, electrolytes, liver and renal function tests, and the renal scintiscan revealed chronic renal insufficiency as a result of poorly regulated diabetes mellitus (Ht = 23,4%, Urea = 206 mg/dl Creatinine = 10,4 mg/dl, Creatinine clearance = 7,5 ml/min, K^+ ^= 6,1 mmol/l, Glucose = 83 mg%, HbA_1_C = 4,5%). Patient's blood glucose's and HbA_1_C values were at the time of admittance normal, as the patient was receiving systematically Glucophage 1 × 2 a month before the admittance. Abdominal ultrasound demonstrated a mass measuring 2.66 × 2.72 cm at the lower pole of the right kidney. This finding was confirmed by abdominal computed tomography (CT) scan which showed a calcified mass, diameter of 2,6 cm, at the lower pole of the right kidney as well as a small mass at the right adrenal gland (Figure 1). The magnetic resonance imaging (MRI) scan also showed a 2,5 × 2,7 cm mass at the lower pole of the right kidney and an adenoma of the right adrenal gland (Figures 2, 3). There was no evidence of lymphadenopathy, involvement of the right renal vein and inferior vena cava or distant metastases. The chest radiography was normal. The patient underwent a transperitoneal laparoscopic right radical nephrectomy. Preoperatively the patient was kept euvolemic, normotensive, normonatremic, normokalemic, not acidotic (pH:7.45) with Ht: 30%. Dialysis was performed within the 24 hours before surgery, which corrected any uremic platelet dysfunction. Sevoflurane was used as a volatile anesthetic. Intraoperatively, invasive monitoring was used to guide fluid therapy. Hypotension and drugs with substantial renal excretion were avoided. Atracurium was preferred as a muscle relaxant, while succinylcholine was avoided. The operative time was 1 hour and 50 minutes. Histological examination showed a Fuhrman grade 2, T1 RCC and a papillary adenoma of the adrenal gland (Figures 4, 5). Postoperatively, nephrotoxic agents, sych as aminoglycosides and nonsteroidal anti-inflammatory drugs (NSAIDS) were avoided. The postoperative period was uneventful, patient's blood urea and creatinine after surgery were 127 mg/dl and 5,7 mg/dl respectively and the patient was discharged on the fourth postoperative day. According to TNM (Tumor, Node, Metastases) staging system, the disease stage was I, so the patient did not receive adjuvant chemotherapy. He is still under hemodialysis and the follow up abdominal CT scan showed no recurrence 6 months postoperatively.

### Technique

**Figure 6:**
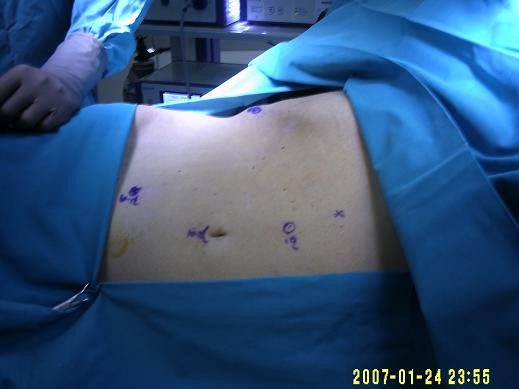
**The patient's position at the operation table and the sites of trocars**.

After general anesthesia was administered, a urinary catheter and a nasogastric tube were placed. The patient was placed in a 45° modified flank position with the top of the iliac crest at the top of the kidney rest and the table flexed. The arms were taped over a folded pillow and the patient was securely taped to the operative table. The initial 10-mm trocar was placed at the umbilicus. Pneumoperitoneum was achieved with a Veress needle to 15 mm Hg at the umbilicus. The 10-mm, 30° laparoscope was used via the umbilical port. A 10-mm trocar was placed midway between the xiphoid and the umbilicus, and a 12-mm trocar was placed lateral to the umbilical port at the edge of the rectus abdominal muscle. A 5-mm trocar was placed in the subxiphoid region and another 10-mm trocar was placed subcostally close to the right anterior axillary line (Figure 6).

The lateral line of Toldt was identified and incised with the harmonic scalpel approximately 1 cm from the colon. The thin layer of peritoneum over the anterior surface of the kidney was mobilized from the iliac vessels to the hepatic flexure of the colon, taking care to avoid entering the Gerota fascia. This plane was bluntly dissected by dividing the colorenal ligament until the colon was rolled medially. Any small vessels that were encountered were controlled with the harmonic scalpel. When the colon had rolled medially, the duodenum was also rolled medially allowing identification of the inferior vena cava.

**Figure 7:**
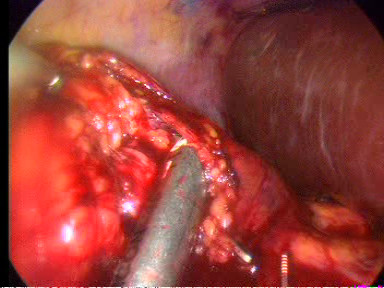
**The en bloc dissection of the renal artery and vein with EndoGIA stapler**.

**Figure 8:**
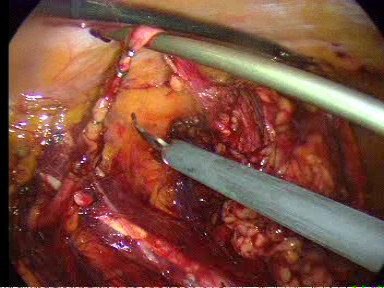
**The transection of the ureter between two 5-mm clips**.

The ureter and gonadal vein were identified and followed superiorly by lateral retraction of them with the 5-mm suction/irrigator. The gonadal vein was dissected using the harmonic scalpel. This manoeuvre allowed identification of the lower pole of the kidney and the renal hilum. The branching of the right gonadal vein from the inferior vena cava was controlled with two 5-mm clips on either side and divided. Then the kidney was retracted anterolaterally, and attachments between the lower pole and the posterior abdominal wall were taken down. Next the kidney was retracted laterally and superiorly to carry out renal hilar dissection. After that the renal vein was identified and dissected circumferentially until enough of its length was free to allow division. The renal artery posterior to the renal vein was also identified and dissected. During the dissection, hilum bleeding was encountered and the renal artery and renal vein were stapled en bloc with the laparoscopic EndoGIA stapler (ENDOPATH^®^, ETS Flex 45 Endoscopic Articulating Linear Cutter 45 mm staple line, 2.5 mm Staple Leg Length (Vascular/Thin). 45 MM Vascular/Thin) with a vascular load (Figure 7). Next the right adrenal vein was identified, dissected and divided between two 5-mm clips on both sides. The medial dissection continued along the inferior vena cava superiorly and subsequently around the adrenal gland. Then the ureter was transected between two 5-mm clips (Figure 8). Finally the kidney was completely dissected by dissecting free the remaining lateral attachments to the body wall and the superior attachments lateral to the adrenal gland.

**Figure 9:**
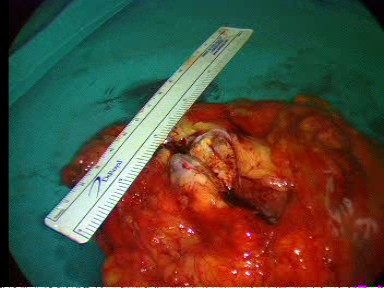
**The specimen of the right kidney revealing the tumor lesion**.

An expanded port site -umbilical- vertical incision was carried out and the 15-mm EndoCatch was placed through a small opening in the peritoneum at the incision site. The kidney with the adrenal gland was placed into the bag, the string was detached and pulled so that the bag closed and the ring that held the bag open was retracted under direct visualization. The opening of the bag was brought out through the incision site. The incision site was approximately 5 cm while the fascia incision was extended to 7 cm (Figure 9). Then the fascia was closed, pneumoperitoneum was re-established, the renal fossa was reexamined and no signs of hemorrhage were encountered. A Robdrain tube No 24 was placed at the right paracolic gutter, which was removed on the 2^nd ^postoperative day. The patient was returned to the supine position and extubated.

## Discussion

The incidence of RCC is higher in dialysis patients than in the general population. Yet, the prognosis is considered to be favorable in these patients, because routine screening may detect the cancers while they are still small [4]. Partial nephrectomy has become the standard treatment for small (<4 cm) renal tumors. In our case the tumor size was just 2,5 × 2,7 cm. However, it is unlikely that patients with end-stage renal disease would benefit from this minimally invasive approach. In fact, satellite tumors apart from the most evident neoplasm are present in approximately 30% of the patients. Therefore, radical nephrectomy is indicated even when the tumor is small [4]. Additionally, in our case, preservation of renal function was not actually a concern, since our patient suffered from chronic kidney disease stage 5 (Kidney failure; creatinine clearance: 8,32 ml/min and GFR: 4,71 mL/min, less than 15 mL/min/1.73 m^2^). It did not seem reasonable to jeopardize the oncologic outcome in an attempt to spare nephrons almost not functioning.

Laparoscopic radical nephrectomy (LRN) increasingly is being performed. The kidney can be approached through the peritoneum or retroperitoneally. The transperitoneal approach is familiar to a general surgeon, with easily identifiable anatomy and a large working space. Therefore we preferred the laparoscopic transperitoneal approach.

Routine removal of the ipsilateral adrenal gland is unnecessary, unless the tumor involves a large portion of the upper pole of the kidney or there is suggestion of adrenal gland abnormality on preoperative staging workup [8]. In our case the preoperative workup revealed a small mass at the right adrenal gland, therefore nephrectomy and adrenalectomy was performed en block.

During the mobilization, care is taken to avoid hemorrhage. Small vessels that are encountered can be cauterized or clipped with a 5-mm clip applier. Alternatively the harmonic scalpel or the LigaSure system may be used to control these vessels. Our general policy is to use the harmonic scalpel when performing advanced laparoscopic surgery. Most surgeons clip and divide the renal artery first and then the renal vein. Alternatively a stapler may be used to divide the renal artery and then the vein. In our case hilum bleeding was encountered, so the renal artery and vein were stapled en bloc with the laparoscopic EndoGIA stapler with a vascular load. We felt this was reasonable in order to avoid serious bleeding and open conversion. Actually, renal artery and vein may be stapled en bloc, when necessary [9]. The right adrenal vein was dissected and divided separately.

The postoperative course was uneventful. The laparoscopic approach was associated with well tolerable postoperative pain, quick oral intake, short hospitalization (3 days), and a rapid recovery and return to previous activities.

## Conclusion

Nephron-sparing surgery has become the treatment of choice for small (<4 cm) renal tumors. However, in patients with end-stage renal disease, radical nephrectomy appears to be a more efficient treatment for localized RCC. Transperitoneal LRN emerges as an oncologically rational procedure for this special situation.

## Consent

Written informed consent was obtained from the patient for publication of this case report and accompanying images. A copy of the written consent is available for review by the Editor-in-Chief of his journal.

## Competing interests

The authors declare that they have no competing interests.

## Authors' contributions

All authors contributed the same.
